# Multi-View Edge Attention Network for Fine-Grained Food Image Segmentation

**DOI:** 10.3390/foods14173016

**Published:** 2025-08-28

**Authors:** Chengxu Liu, Guorui Sheng, Weiqing Min, Xiaojun Wu, Shuqiang Jiang

**Affiliations:** 1 School of Information and Electrical Engineering, Ludong University, Yantai 264025, China; chengxuliu@m.ldu.edu.cn (C.L.); shengguorui@ldu.edu.cn (G.S.); 2 The Key Laboratory of Intelligent Information Processing, Institute of Computing Technology, Chinese Academy of Sciences, Beijing 100190, China; sqjiang@ict.ac.cn; 3 University of Chinese Academy of Sciences, Beijing 100049, China; 4 Institute of Science & Technology, Jiangnan University, Wuxi 214122, China; wu_xiaojun@jiangnan.edu.cn

**Keywords:** food image, image segmentation, food health, deep learning

## Abstract

Precisely identifying and delineating food regions automatically from images, a task known as food image segmentation, is crucial for enabling applications in food science such as automated dietary logging, accurate nutritional analysis, and food safety monitoring. However, accurately segmenting food images, particularly delineating food edges with precision, remains challenging due to the wide variety and diverse forms of food items, frequent inter-food occlusion, and ambiguous boundaries between food and backgrounds or containers. To overcome these challenges, we proposed a novel method called the Multi-view Edge Attention Network (MVEANet), which focuses on enhancing the fine-grained segmentation of food edges. The core idea behind this method is to integrate information obtained from observing food from different perspectives to achieve a more comprehensive understanding of its shape and specifically to strengthen the processing capability for food contour details. Rigorous testing on two large public food image datasets, FoodSeg103 and UEC-FoodPIX Complete, demonstrates that MVEANet surpasses existing state-of-the-art methods in segmentation accuracy, performing exceptionally well in depicting clear and precise food boundaries. This work provides the field of food science with a more accurate and reliable tool for automated food image segmentation, offering strong technical support for the development of more intelligent dietary assessment, nutritional research, and health management systems.

## 1. Introduction

Driven by increasing public health awareness and the growing demand for scientific dietary planning, the ability to automatically and precisely identify and separate food regions from images has become a critical technical requirement in the field of food science [1]. This technique, commonly referred to as food image segmentation, serves as an essential foundation for applications such as automated dietary tracking, accurate nutritional analysis, and food safety supervision. Its main objective is to meticulously delineate the specific contours of each food item within complex images containing food, utensils and backgrounds [2]. Only with such precise food contour information can subsequent processes like automatic food recognition [3,4,5,6,7], nutritional content calculation [8,9,10,11], and personalized health management recommendations [12,13,14] become truly reliable. Despite recent advances in related technologies, achieving high-precision food contour extraction in real-world scenarios (e.g., stacked food items, blurred boundaries, and diverse food categories) still poses significant challenges. Therefore, the development of more accurate and robust food image segmentation methods has substantial research value and broad application prospects to advance nutrition research, develop intelligent health applications, and improve the efficiency of food safety monitoring.

However, food image segmentation is fraught with the following distinct challenges: (1) High Intra-Class Variance: A single food item can exhibit vastly different appearances due to varied preparation and cooking methods (e.g., steaming, stir-frying, stewing). This challenge is particularly pronounced in complex cuisines, such as Chinese food, and is further exacerbated when ingredients possess irregular shapes or lack distinctive color and texture features. (2) Ambiguous Boundaries: The delineation between different food items or between food and the background is often ill-defined. For instance, a serving of rice, composed of numerous small, individual components, exemplifies this issue. Although the overall region is discernible, the scattered nature of individual grains impedes the precise definition of a compact boundary.

Recent years have witnessed significant progress in food image segmentation [15], primarily driven by rapid advancements in machine learning and computer vision. Early innovations, such as Fully Convolutional Networks (FCNs), pioneered end-to-end pixel-level classification [16]. Subsequent architectures introduced critical enhancements; for example, dilated convolutions [17] expanded the receptive field without compromising spatial resolution, and the Pyramid Scene Parsing Network (PSPNet) aggregated multi-scale contextual information through diverse pooling layers [18]. To better capture long-range dependencies, non-local methods were developed to model relationships between pixel pairs across feature maps [19], a concept later refined with cross-attention mechanisms for improved computational efficiency [20].

More recently, Transformer-based architectures [21,22] have instigated a paradigm shift. Vision Transformers (ViTs) have been effectively applied to food image segmentation, more proficiently leveraging global contextual information to enrich feature representations and yield superior segmentation results [23,24]. The advent of the Segment Anything Model (SAM) has also provided a powerful tool, subsequently adapted and fine-tuned for improved performance on food datasets [25,26,27]. Furthermore, dedicated efforts in food-specific domains have shown notable advancements. For instance, Wu et al. [28] developed ReLeM, the first pretraining model for food images, which integrates recipe information with visual features to mitigate intra-class variance. Currently, generative techniques have been explored; Jaswanthi et al. [29] proposed a hybrid method that uses generative adversarial networks (GANs) [30] to produce auxiliary masks for CNN-based classification. Generic segmentation and detection methods have also found applications in food image segmentation [31,32,33]. The burgeoning field of Large Language Models (LLMs) is also poised to offer novel, multimodal solutions for food image segmentation [34,35].

Despite these substantial advancements, many existing models still operate at a coarse granularity, often resulting in imprecise segmentation, particularly evident at the fine-grained edges and boundaries of food items.We hypothesize that standard foundation models like SAM, while powerful, produce suboptimal results on fine-grained segmentation tasks because their decoders lack sufficient domain-specific detail from the early-stage features. We posit that by explicitly engineering a multi-view feature extraction pipeline to capture and fuse complementary local and global information, we can create an enriched feature representation. This enhanced representation, when integrated with a detail-focused decoder, will empower the model to more accurately delineate complex food boundaries compared to architectures that lack this targeted, synergistic feature enhancement. To address these limitations, our paper introduces the Multi-view Edge Attention Network (MVEANet), a novel food image segmentation method. Built upon the Segment Anything Model (SAM) [27], MVEANet integrates a multi-view feature fusion mechanism, inspired by Multi-view Aggregation Network(MVANet) [36], and incorporates High-Quality Tokens (HQ-Tokens) [37] to significantly improve the prediction of fine-grained mask details. The code is available at https://github.com/Axboexx/MVEANet (accessed on 20 August 2025).

## 2. Materials and Methods

### 2.1. Datasets

**Figure 1:**
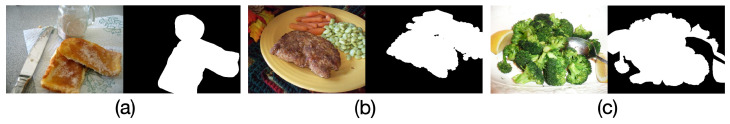
(**a**–**c**) are food images from the FoodSeg103 dataset alongside their corresponding ground-truth semantic segmentation masks.

We evaluate our method using the following publicly available food image segmentation datasets:

**FoodSeg103** [28]: This dataset was constructed based on the Recipe1M dataset [38]. Initially, the most frequent ingredient categories from Recipe1M were identified, and the top 124 were selected. After a further screening process to ensure class distinction and quality, this set was refined to a final 103 ingredient categories. The creators then selected images from Recipe1M that contained between 2 and 16 distinct and clearly annotatable ingredients. This selection process yielded a final dataset of 7118 images with corresponding pixel-level masks. Figure 1 shows several examples from this dataset.

**Figure 2:**
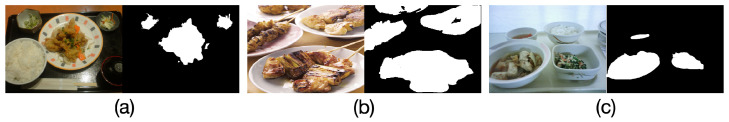
(**a**–**c**) are food images from the UEC-FoodPIX Complete dataset alongside their corresponding ground-truth semantic segmentation masks.

**UEC-FoodPIX Complete** [39]: This large-scale dataset is a direct quality enhancement of the original UEC-FoodPix. It contains 10,000 images covering 102 dish categories. The key contribution of the “Complete” version is the meticulous, manual refinement of the segmentation masks; while the masks in the training set of original dataset were generated semi-automatically with the GrabCut algorithm, leading to boundary inaccuracies, all masks in this version have been corrected by human annotators following a strict set of predefined rules to ensure high precision. Example images are provided in Figure 2.

### 2.2. Equipment and Experimental Setup

The operating system version is Ubuntu 20.04 LTS. We use Pytorch 1.12.0 [40] and Python 3.8 to construct our model, which is then trained on an NVIDIA A800 GPU (80 GB), an Intel(R) Xeon(R) Platinum 8358 CPU @2.60 GHz, 8 GB RAM, and a 1TB SSD. During the training process, for the FoodSeg103 dataset, 4983 images are used as the training set, along with 4983 corresponding training mask images, and 2135 images with 2135 corresponding mask images for testing. The image sizes are resized to 1024×1024, and the batch size is set to 1. For the UEC-FoodPIX Complete dataset, 9000 images are selected for training and 1000 images for testing. The image sizes are also resized to 1024×1024, and the batch size is set similarly to 1.

### 2.3. Method

**Figure 3:**
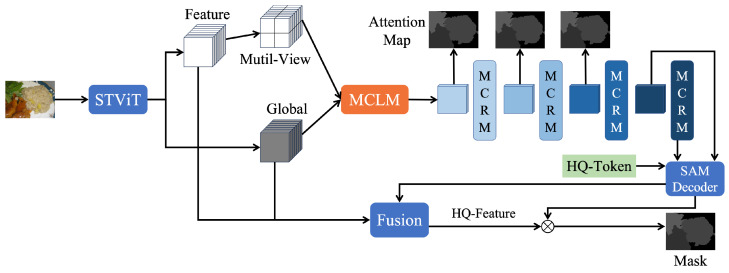
Overall architecture of the MVEANet. STViT acts as the backbone, extracting global features and creating distant views. MCLM and MCRM process these features further, generating multiple intermediate prediction masks. The decoder produces the prediction mask. During training, we optimize the loss function using both the intermediate masks and the final prediction mask, but for testing, only the final prediction mask is output.

To validate the efficacy of multi-view and HQ-Token integration for food image segmentation, this paper introduces the Multi-view Edge Attention Network (MVEANet). The overall architecture of the model is illustrated in Figure 3, primarily divided into three parts: The first part employs Super Token Vision Transformer(STViT) [41] as its backbone network for rapid global feature extraction and to generate the distant view of input data. The second part is the feature extraction module, where we utilize the feature extraction module of MVANet [36] to further process the input data from the first part and generate multiple intermediate prediction masks. The third part is the HQ-SAM Decoder, which fuses the HQ-Token [37] with the SAM decoder [27] to output the final segmentation result for the input image. Notably, during model optimization, the multiple intermediate masks generated by the second part, along with the final segmentation result, are simultaneously fed into the loss function for optimization. In the testing phase, only the predicted mask generated by the third part is output.

We adopt the loss function configuration from MVANet [36]. The total loss function *L* is an aggregation of losses from intermediate representation and the final prediction map, as described in the paper. Intermediate representation include local representation, global representations and attention maps, denoted as $l_{l},l_{g},l_{a}$, respectively. The final prediction map is represented as $l_{f}$. Loss *l* employs the combination of the binary cross-entropy (BCE) loss and the weighted IoU loss, a common practice in segmentation tasks. Its definition is as follows:

(1) $$l=l_{BCE}+l_{IoU}$$

The total loss *L* is therefore defined as follows:

(2) $$L=l_{f}+\sum_{i=1}^{5}\left(l_{l}^{i}+\lambda_{g}l_{g}^{i}+\lambda_{a}l_{a}^{i}\right).$$

Among them, $\lambda_{g}$ and $\lambda_{a}$ are weighting coefficients, and we also keep the value of 0.3.

#### 2.3.1. STViT Backbone

**Figure 4:**
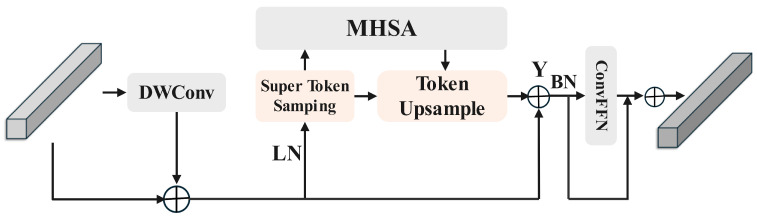
Architecture of the Super Token Vision Transformer (STViT); Super Token Sampling for Efficient Vision Transformers.

Our model employs STViT [41] as its backbone. STViT is a general-purpose Vision Transformer designed to address the high computational complexity of the self-attention mechanism in traditional Vision Transformers. The core of STViT lies in its proposed Super Token Attention (STA) mechanism, which comprises three processes: Super Token Sampling (STS), Multi-Head Self-Attention (MHSA), and Token Upsampling (TU). In particular, STS reduces complexity through iterative steps and sparse computation, where, for each token, only its surrounding 3×3 superpixels are used to compute associations. The structure of the basic STViT module, the Super Token Transformer Block, is shown as Figure 4.

#### 2.3.2. Feature Extraction

**Figure 5:**
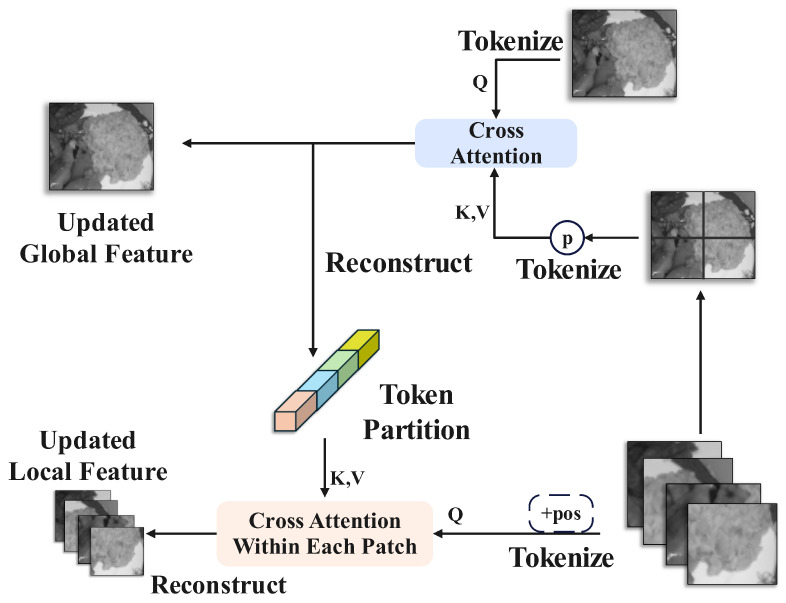
Architecture of the Multi-view Complementary Localization Module (MCLM), using multi-grained pooling and cross-attention to achieve complementary localization of global and local features.

**Figure 6:**
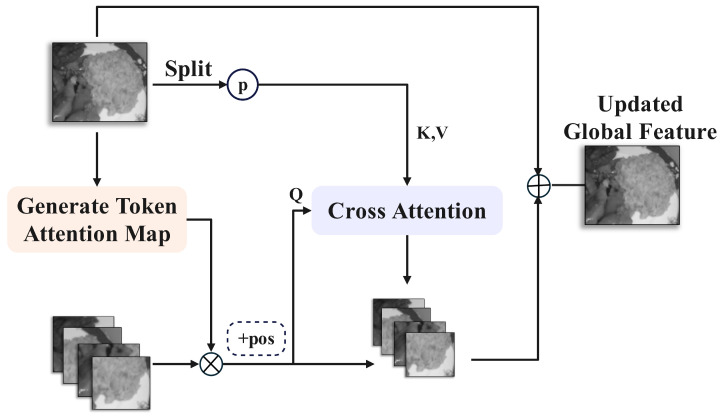
Architecture of the Multi-view Complementary Refinement Module(MCRM), refining global features using detailed local information and boosting multi-view complementarity via cross-attention.

Conventional image segmentation methods typically proceed directly to decoding after encoding by a backbone model or encoder. However, given that food images are fine-grained and present greater segmentation challenges than general images, we introduce a new set of feature extraction methods between the encoder and decoder. These methods are derived from the Multi-view Complementary Localization Module (MCLM) and Multi-view Complementary Refinement Module (MCRM) proposed in MVANet [36]. MCLM aims to achieve complementary localization of global and local features through multi-grained pooling and cross-attention. MCRM utilizes the detailed information from local features to refine global features and enhances multi-view complementarity through cross-attention. The structures of MCLM and MCRM are shown in Figure 5 and Figure 6, respectively.

After processing in Section 2.3.1, multi-level feature maps are generated and denoted $E_{i}|i=1,2,3,4,5$. Among these, $E_{5}$ represents the panoramic view, while $E_{1}\;E_{4}$ corresponds to the local views. First, the map of features $E_{5}$ is divided into a global feature $E_{5}^{G}\in R^{B\times C\times \frac{H}{32}\times \frac{W}{32}}$ and a set of local features ${\left\{E_{5}^{L_{m}}\right\}}_{m=1}^{M}$, where $E_{5}^{L_{m}}\in R^{B\times C\times \frac{H}{32}\times \frac{W}{32}}$. Subsequently, aligned with their respective positions in the original image, these local features are assembled into unified global features $E_{5}^{L_{9}}\in R^{B\times C\times \frac{H}{32}\times \frac{W}{32}}$. Following this, multi-grained pooling is used to generate pyramid features:

(3) $$P_{n}={\mathrm{AvgPool}}_{n}\left(E_{5}^{L_{g}}\right),\;n\in \left\{1,2,\ldots ,N\right\},$$

where $E_{5}^{L_{g}}$ is the unified global feature, and *N* denotes the number of parallel pooling branches. Subsequently, cross-attention is performed between the global features and the multi-grained features.

(4) $$T^{G}=T\left(E_{5}^{G}\right)+\mathrm{LN}\left(\mathrm{MHCA}\left(T\left(E_{5}^{G}\right)W^{Q},\left[T\left(P_{1}\right),\ldots ,T\left(P_{N}\right)\right]W^{K,V},\left[T\left(P_{1}\right),\ldots ,T\left(P_{N}\right)\right]W^{K,V}\right)\right).$$

Here, T(·) signifies the tokenization operation, $W^{Q}$ and $W^{K,V}$ are projection matrices, MHCA refers to Multi-Head Cross-Attention, LN is Layer Normalization, and FFN is the Feed-Forward Network. This is immediately followed by cross-attention between the local features and the global tokens,

(5) $$T_{m}^{L^{\prime }}=\mathrm{MHCA}\left(T\left(E_{5}^{L_{m}}\right)W_{m}^{Q},T^{G_{m}},T^{G_{m}}\right),$$

where $E_{5}^{L_{m}}$ is the m−th local feature, and $T^{G_{m}}$ corresponds to the portion of the rearranged global token aligning with the local region. Finally, feature fusion is performed to generate the feature map for subsequent processing.

(6) $$D_{5}=\left[E_{5}^{G^{\prime }},{\left\{E_{5}^{L_{m}^{\prime }}\right\}}_{m=1}^{M}\right].$$

The MCRM (Multi-view Complementary Refinement Module) takes input features denoted as $D_{i}$, where i∈{1,2,3,4,5} represents the layer index. Similar to MCLM, the feature $D_{i}$ is partitioned along the batch dimension into $D_{i}^{G}$ and ${\left\{D_{i}^{L_{m}}\right\}}_{m=1}^{M}$ before processing,

(7) $$A=\mathrm{sigmoid}(\mathrm{conv}\left(D_{i}^{G}\right)),$$

where $D_{i}^{G}$ is the global feature, $D_{i}^{L_{m}}$ is the local feature, *A* is the attention map, ⊙ denotes the Hadamard product, and assemble and split are operations for combination and decomposition of features, respectively. Subsequently, a multi-grained pooling process similar to that in MCLM is applied to ${\left\{D_{i}^{L_{m}}\right\}}_{m=1}^{M}$ to obtain multi-perceptual tokens $T_{i}^{G_{M}}$ with different contextual information for the m−th patch. These tokens are concatenated to serve as the *K* and *V* for cross-attention, followed by the cross-attention operation.

(8) $$T_{i}^{L_{m}}=\mathrm{MHCA}\left(T\left(D_{i}^{L_{1}}\right),\ldots ,T\left(D_{i}^{L_{M}}\right)\right)W^{Q_{i}},\left[T_{i}^{G_{1}},\ldots ,T_{i}^{G_{M}}\right]W^{K_{i},V_{i}},\left[T_{i}^{G_{1}},\ldots ,T_{i}^{G_{M}}\right]W^{K_{i},V_{i}}.$$

Finally, refined feature fusion is performed as follows:

(9) $$D_{i}^{G^{\prime }}=D_{i}^{G}+\mathrm{sum}\left({\left\{D_{i}^{L_{m}^{\prime }}\right\}}_{m=1}^{M}\right),$$

(10) $$D_{i}^{\prime }=\left[{\left\{D_{i}^{L_{m}^{\prime }}\right\}}_{m=1}^{M},D_{i}^{G^{\prime }}\right],$$

where $D_{i}^{L_{m}^{\prime }}$ represents the reconstructed local features from the updated local tokens, and $D_{i}^{G^{\prime }}$ is the detail-enhanced globally optimized feature.

#### 2.3.3. Detail Enhancement Decoder

To address the issue of insufficient mask quality often encountered in traditional segmentation models when dealing with complex structures and fine boundaries, HQ-SAM [37] introduced the innovative concept of a High-Quality Output Token (HQ-Token). The core idea behind the HQ-token is to enable the model to generate higher-quality segmentation masks without significantly increasing the complexity or computational cost of the model. Specifically, the HQ-token is designed as a special, learnable token injected into the mask decoder. It not only operates on intrinsic features of the decoder, but, more crucially, it can effectively fuse features extracted from the early and final layers of the backbone network (typically low-level and high-level features from a Vision Transformer). This fusion mechanism allows the HQ-Token to simultaneously capture both global contextual information and fine-grained local details from the image. Through this approach, the HQ-Token guides the model during the decoding process to pay closer attention to the precision of object boundaries, the integrity of internal structures, and the expressiveness of details, thereby significantly enhancing the overall quality of generated masks and mitigating common artifacts, holes, or unsmooth boundary issues.

## 3. Results

This section will detail the performance of MVEANet in the FoodSeg103 and UEC-FoodPIX Complete, including a comparison with other segmentation models. Subsequently, we will present the setup and results of our ablation studies, validating the positive contribution of each component of MVEANet to the segmentation results. Finally, we will present the qualitative results of our proposed method in the food segmentation task.

**Evaluation Metrics.** Mean Absolute Error (MAE) quantifies the average pixel-wise absolute difference between a continuous prediction map and a binarized ground truth mask (gt). Here, *W* and *H* represent the width and height of the image, respectively. Lower MAE indicates superior performance. $F_{\beta }^{max}$ and $F_{\beta }^{\omega }$ are the maximum and weighted scores of precision and recall, respectively, where $\beta^{2}$ is set to 0.3. $S_{m}$ concurrently assesses the structural similarity between the prediction and the mask, considering both the characteristics of region-level and object-level. $E_{\emptyset }^{m}$ is widely used for evaluating the correspondence of pixel-level and image-level. Mean Intersection over Union(mIoU) measures the overlap between the prediction and the ground truth.

### 3.1. Comparative Experimental Results

Our proposed model establishes a new state-of-the-art (SOTA) in food image segmentation, consistently outperforming existing methods across two benchmark datasets. As comprehensively detailed in Table 1 and Table 2, our model exhibits superior performance across a multitude of evaluation metrics. Specifically, it enhances the mIoU of MVANet by 1.6% on the UEC-FoodPix Complete and by a more substantial 4.6% on the FoodSeg103.

### 3.2. Ablation Study

To comprehensively evaluate the impact of various backbone architectures on model performance, we conducted a comparative analysis. The experiment was designed to evaluate the effectiveness of different backbones in designated tasks, their generalizability and their computational efficiency, measured in Frames Per Second (FPS). First, we perform 10 untimed inference iterations as a warm-up to eliminate interference from irrelevant factors. We then measure the total time of 100 consecutive inference runs. The final FPS is calculated as the average of these 100 runs. We synchronize the GPU computation flow before and after the timing loop to ensure accurate measurement of the GPU execution time.

A critical aspect of our study was ensuring our proposed model is not only accurate but also computationally efficient, a key requirement for practical applications. To this end, we conducted a quantitative analysis of the trade-off between performance and speed across several backbone architectures, with results presented in Table 3. This study serves as our primary investigation into the efficiency of the model.

The results show that while the Swin-Transformer [51] backbone achieves the highest accuracy, it does so at a significant computational cost (5.8 FPS). In contrast, STViT [41] provides a much more compelling balance, delivering strong segmentation performance (0.693 mIoU on FoodSeg103) at a faster inference speed (6.3 FPS). Therefore, we selected STViT [41] as the final backbone for MVEANet, as it represents the best compromise between high accuracy and practical efficiency. This FPS comparison provides a direct, practical measure of the end-to-end computational cost of each configuration, informing our final architectural design.

To further validate the effectiveness of each proposed component within our model, we performed an in-depth ablation study, with the results concisely summarized in Table 4. Our model integrates four principal design elements: a Multi-View strategy, MCLM, MCRM, and the HQ-Token. This study systematically quantifies the individual contribution of each component through a controlled incremental analysis. Our ablation study, summarized in Table 4, systematically deconstructs the impact of each major component of our network. The baseline, which utilizes the Multi-View without MCLM or MCRM, achieves a foundational mIoU of 0.594. Adding only MCLM yields a significant performance increase to 0.641 mIoU. The key takeaway here is that MCLM is highly effective at its primary task: improving object localization. By using global tokens to guide the local feature patches, it helps the method to correctly identify where the food items are within the detailed close-up views and filter out background noise. Conversely, adding only MCRM also increases performance to 0.633 mIoU. This demonstrates a distinct contribution of MCRM: enhancing fine-grained details. It excels at using detailed information from local views to refine the texture and boundaries of object masks when both methods are used together, achieving an mIoU of 0.652. This result is greater than what would be expected from simply summing their individual improvements, highlighting a clear synergy. This synergy arises because the methods perform complementary and sequential tasks: MCLM first provides a clean, well-localized feature map, which then allows MCRM to apply its powerful detail refinement capabilities far more effectively. Building on this strong foundation, HQ-Token further improves mIoU to 0.667. HQ-Token acts as a specialized tool in the decoder stage; it is specifically designed to translate the high-quality, refined features produced by our MCLM-MCRM pipeline into a final segmentation mask. For performance evaluation, we used MAE and mean mIoU as key metrics. A comparative analysis of the performance data across these diverse configurations unequivocally demonstrates two key findings: (1) Each of our proposed modules contributes a discernible and tangible performance gain, and (2) the synergistic interaction among all modules culminates in the optimal performance achieved by our final model.

### 3.3. Qualitative Evaluation

**Figure 7:**
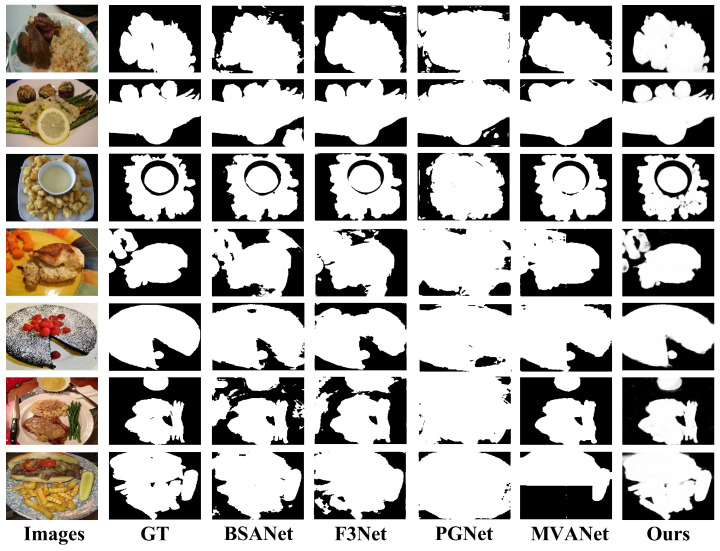
Segmentation results of the models in UEC-FoodPix Complete.

Figure 7 presents the qualitative segmentation results of our proposed model along with other mainstream methods in selected samples from UEC-FoodPix Complete. Compared with ground truth, it can be clearly seen that our model shows significant advantages in the completeness of the generated segmentation mask and the accuracy of edge details. For example, when dealing with food with complex and irregular shapes as shown in the second and seventh rows, PGNet tends to produce large areas of wrong segmentation (over-segmentation), while BSANet and $F^{3}$Net fail to capture the entire food area. Although the results of MVANet are relatively good, there are still obvious details lost and adhesion problems when dealing with the details of the food in the bowl in the fourth row and the boundaries of the food in the fifth row. In contrast, our model can accurately outline the contours of food, effectively distinguish different food entities, and retain key internal details. Its segmentation results are visually closest to the ground truth.

**Figure 8:**
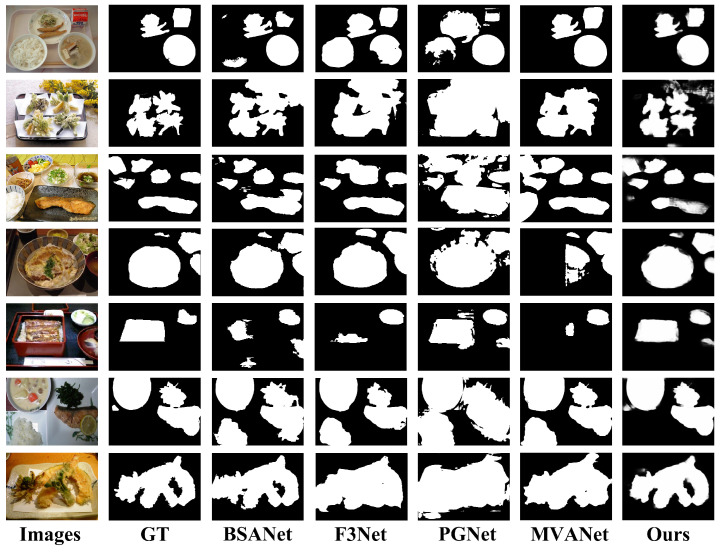
Segmentation results of the models in FoodSeg103.

To further verify the generalization and robustness of our model, Figure 8 shows the qualitative comparison results on the FoodSeg103 with more diverse scenes. The results once again confirm the superiority of our model. A very convincing example is the ring-shaped food in the third row: all other models, especially PGNet and MVANet, failed to identify the empty area in the center of the food. Our method correctly distinguishes food from background. In addition, when processing the cake in the fifth row and the multi-object scene in the sixth row, our method is able to generate better edges than other methods and better separate neighboring foods (such as chicken and asparagus). These visualization results strongly demonstrate that compared to existing methods, our model has a stronger ability to handle complex spatial layouts, maintain detail integrity, and accurately locate boundaries.

## 4. Discussion

Despite the promising performance of MVEANet in the FoodSeg103 and UEC-FoodPIX Complete datasets, several avenues of improvement remain. Firstly, the current backbone network could potentially be replaced by more specialized alternatives, such as models specifically designed for food computing or those extensively pretrained on large-scale food datasets. Second, we observed that the performance of the model is degraded when segmenting foods with amorphous boundaries, heavily mixed ingredients, or significant overlap, such as in stews or mixed salads. We hypothesize that purely visual approaches face inherent limitations in these ambiguous scenarios. Therefore, a promising direction for future research is to explore multimodal solutions, such as integrating textual information like recipes or ingredient lists, to provide the necessary contextual priors to resolve these visual ambiguities. Finally, with rapid advancements in large models, there is significant potential to support food image segmentation, as their superior image understanding and generation capabilities can provide even more effective feature information for segmentation tasks.

## 5. Conclusions

Multi-view Edge Attention Network (MVEANet) addresses limitations in fine-grained food image segmentation, particularly concerning imprecise boundaries and diverse appearances, common in previous models. This SAM-based method integrates multi-view and HQ-Token, utilizing an STViT backbone for global feature extraction. MCLM and MCRM of MVANet provide complementary localization and refine features, while the HQ-Token enhances mask quality by fusing multi-level features for accurate boundary depiction. We validate the effectiveness of MVEANet using the FoodSeg103 and UEC-FoodPIX Complete datasets. This approach aims to improve the localization and boundary delineation of pixels, allowing applications such as nutritional assessment to be improved. Future work will focus on quantitative analysis, generalizability, and real-time performance.

## Figures and Tables

**Table 1:** Comparison with other segmentation methods on UEC-FoodPix Complete. The dataset contains 10,000 images covering 102 dish categories. ↓ represents the lower value is better, while ↑ represents the higher value is better.

Method	Evaluation Metrics					
	MAE↓	$F_{\beta }^{max}↑$	$F_{\beta }^{\omega }↑$	$S_{m}↑$	$E_{m}↑$	mIoU↑
$F^{3}$Net [42]	0.281	0.779	0.598	0.681	0.639	0.572
GCPANet [43]	0.194	0.807	0.626	0.729	0.662	0.627
PFNet [44]	0.259	0.776	0.483	0.618	0.624	0.317
BSANet [45]	0.295	0.759	0.535	0.661	0.607	0.437
IFA [46]	0.321	0.726	0.503	0.636	0.585	0.420
PGNet [47]	0.158	0.819	0.674	0.738	0.653	0.631
ISNet [48]	0.264	0.784	0.612	0.706	0.651	0.604
UDUN [49]	0.163	0.822	0.663	0.747	0.685	0.647
FP-DIS [50]	0.163	0.832	0.653	0.741	0.668	0.628
MVANet [36]	0.153	0.867	0.679	0.778	0.708	0.652
Our	0.131	0.886	0.699	0.796	0.743	0.668

**Table 2:** Comparison with other segmentation methods on FoodSeg103. The dataset consists of 7118 images across 103 ingredient categories. ↓ represents the lower value being better, while ↑ represents the higher value being better.

Method	Evaluation Metrics					
	MAE↓	$F_{\beta }^{max}↑$	$F_{\beta }^{\omega }↑$	$S_{m}↑$	$E_{m}↑$	mIoU↑
$F^{3}$Net [42]	0.268	0.698	0.559	0.653	0.519	0.526
GCPANet [43]	0.249	0.718	0.598	0.681	0.557	0.556
PFNet [44]	0.303	0.769	0.497	0.643	0.503	0.465
BSANet [45]	0.270	0.689	0.538	0.649	0.508	0.486
IFA [46]	0.279	0.668	0.528	0.638	0.498	0.478
PGNet [47]	0.237	0.721	0.628	0.698	0.565	0.574
ISNet [48]	0.253	0.712	0.562	0.677	0.532	0.529
UDUN [49]	0.201	0.749	0.653	0.712	0.581	0.607
FP-DIS [50]	0.201	0.756	0.664	0.702	0.564	0.585
MVANet [36]	0.187	0.763	0.676	0.754	0.619	0.647
Our	0.158	0.786	0.722	0.758	0.718	0.693

**Table 3:** Ablation experiments of different backbones on UEC-FoodPix Complete and FoodSeg103. ↑ represents the higher value being better.

Method	FPS	UEC-FoodPix Complete	FoodSeg103
		mIoU↑	mIoU↑
Swin-Transformer [51]	5.8	0.681	0.703
SAM-Encoder [27]	4.9	0.610	0.629
CAS-ViT [52]	8.6	0.532	0.496
STViT [41]	6.3	0.668	0.693

**Table 4:** Ablation experiments of each component. ↓ represents the lower value is better, while ↑ represents the higher value is better.

Multi-View	MCLM	MCRM	HQ-Token	UEC-FoodPIX Complete	
				MAE↓	mIoU↑
	✓	✓	✓	0.179	0.594
✓			✓	0.171	0.641
✓	✓	✓		0.173	0.633
✓		✓	✓	0.169	0.652
✓	✓		✓	0.163	0.667
✓	✓	✓	✓	0.158	0.693

## Data Availability

The original contributions presented in the study are included in the article, further inquiries can be directed to the corresponding author.
